# Outing

**Published:** 1886-08

**Authors:** 


### *OUTING.*

This is the season of *outing*, as the Summer tours and excursions from our crowded business centres have come to be termed. Almost everyone who has the time and means at command, now forsakes his pent up city habitation for the freer, wider and healthier allurements of travel or the less active recreations attendant upon a temporary home in the country, which, owing to the prevalence of gentle airs and refreshing showers, wears to-day all the freshness and beauty of early June.

There is scarcely a district to be found of easy access which accommodates itself to all tastes, in a degree equal to that which lies adjacent to the vast populations of which New York forms the centre. Upon either hand both land and sea offer their allurements. A fondness of adventure in wild, uninhabited regions, naturally suggests the Adirondacks. Here are bold uplands holding aloft their crown of pines, and untamed valleys which stoop to the tangled margin of sparkling lakes, where gun and rod are sure to reach their prey.

In the settled districts whithersoever one directs his course, are well-known summer resorts, ranging all the way from the ample hotel, swarming with life and fashion, to the quieter and less obtrusive farm-house where fresh eggs and real milk are an everyday affair. There, too, are open fields for health-giving rambles, shady nooks and leafy brooksides for the inert and ease-loving; and still nearer at hand, grassy lawns, leveled and cropped to the most fastidious requirement, where lawn tennis and croquet, hold almost hourly court, with bewitching contrasts of gay costumes, and still gayer assemblages of youth and beauty, radiant amid the natural and graceful surroundings of peaceful landscapes, with their ever varying hues of silvery sunlit leaves and glossy greens interspersed with buds of carefully cultured flowers, of which the moving figures seem not as things apart, but parts of a whole in its completeness which it would seem a sacrilege to dissassociate.

We welcome the *outing period*, as the best of physicians and the truest of friends to all classes of people, but especially to the "weary and heavy laden" of this work-a-day world.

"On the sea-beaten shore—'mid the dwellings of men—
In the field or the forest, or wild mountain glen;
Wherever the grass or a daisy could spring,
Or the musical laughter of childhood could ring;
Wherever a swallow could build neath the eaves,
Or a squirrel could hide in his covert of leaves,
I have felt the sweet presence, and heard the low call
Of the *Spirit of Natnre*, which quickens us all."

